# Ganglioneuroma of the External Auditory Canal and Middle Ear

**DOI:** 10.1155/2017/4736895

**Published:** 2017-08-10

**Authors:** Hesham Saleh Almofada, Michael Steven Timms, M. Anas Dababo

**Affiliations:** ^1^Department of Otolaryngology-Head and Neck Surgery, King Faisal Specialist Hospital and Research Center, Riyadh, Saudi Arabia; ^2^King Faisal Specialist Hospital and Research Center, Riyadh, Saudi Arabia

## Abstract

**Objective:**

We report an extremely rare case of ganglioneuroma involving the external auditory canal and middle ear.

**Case Report:**

Ganglioneuromas are rare benign mature tumors thought to originate from sympathetic ganglions, with the highest incidence in the retroperitoneum, adrenal medulla, and posterior mediastinum. We present a case of ganglioneuroma of the external auditory canal and middle ear. At the age of 12 months, the patient was diagnosed with neuroblastoma stage IV with metastasis to the squamous temporal bone, bone marrow, and skull base. He received a high-risk protocol regimen resulting in complete remission. The patient later presented with recurrent right ear discharge at the age of six years and was diagnosed with ganglioneuroma of external auditory canal and middle ear after appropriate investigations. We report in this article the clinical presentation, investigations, surgical intervention, and follow-up.

**Conclusion:**

After the literature review and to our knowledge, this is the first reported case of its kind. Ganglioneuroma maturing from neuroblastoma is one of the theories describing pathophysiology of the disease. Ganglioneuroma should be considered in the differential diagnosis of patients presenting with recurrent ear discharge and decreased hearing in treated cases of neuroblastoma with metastases to temporal bone.

## 1. Introduction

Ganglioneuromas are tumors originating from the autonomic nervous system ganglion cells. They are slow-growing benign tumors derived from the neural crest cells, which are found at any location where the sympathetic tissues are normally found [1]. The most common sites of presentation are the adrenal medulla, retroperitoneum, and posterior mediastinum. Rarely do they present in the head and neck region [2–6]. Ganglioneuromas tend to present late as catecholamine and steroid hormones are not released and due to the protracted growth period [1, 3, 4, 7]. Histologically, their primary features are neuroblasts, ganglion cells, Schwann cells, and stroma. Other features include necrosis, mitosis, hemorrhage, fibrosis, lymphocytic infiltration, and karyorrhexis [1, 2]. They are usually discovered when they start to affect the cranial nerves and sympathetic function or incidentally by imaging done for other reasons [1–4].

## 2. Case Report

**Figure 1:**
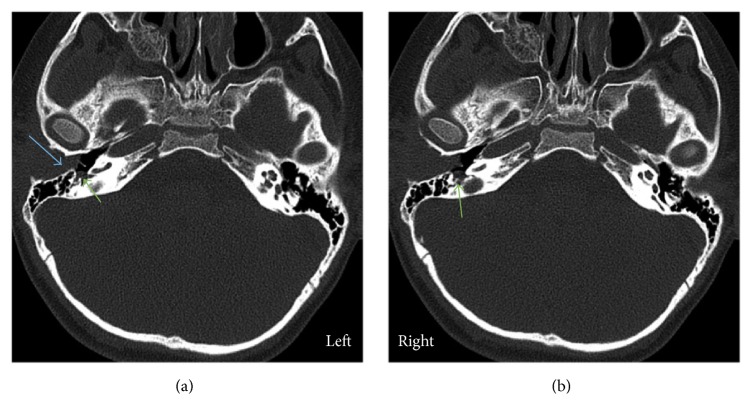
Axial CT scan. Showing soft tissue mass in the right external auditory canal with widening and occlusion of right ear canal (blue arrow) and small middle ear soft tissue opacity (green arrow).

**Figure 2:**
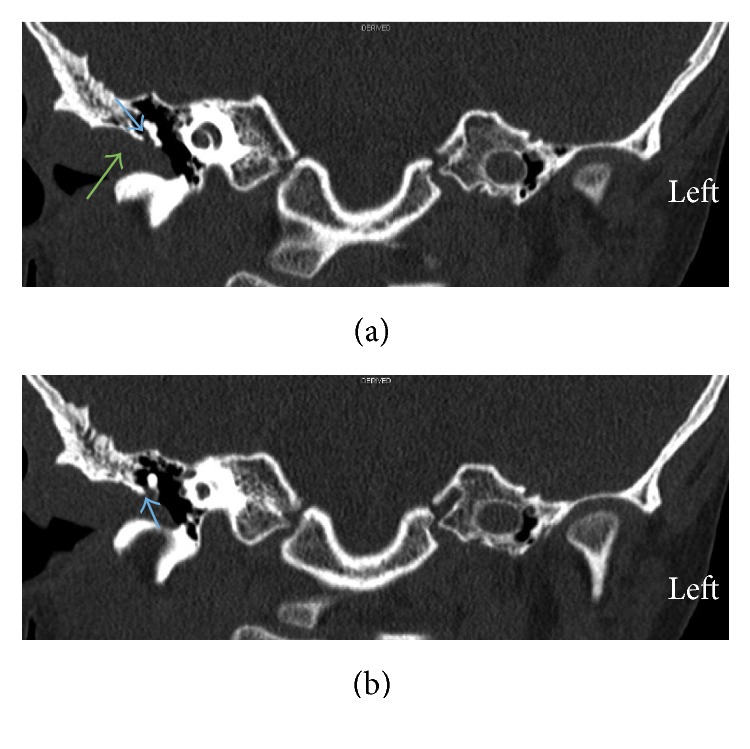
Coronal CT scans. (a) Soft tissue mass in the right external auditory canal (green arrow) extending into Prussak's space (blue arrow). (b) Minimal erosion of the scutum (blue arrow).

**Figure 3:**
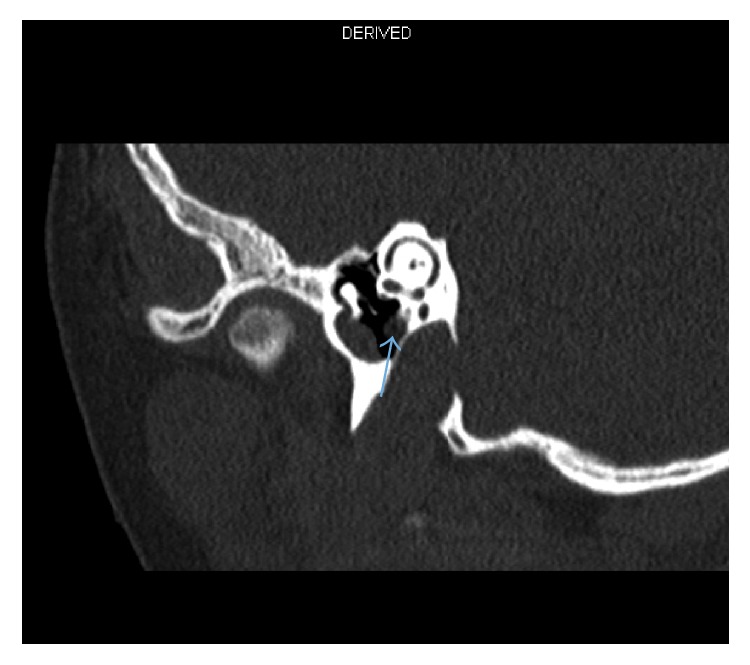
Limited coronal CT scan of right ear revealing a lesion in the area of the second genu of the facial nerve (blue arrow).

At 12 months of age, our patient was referred to our center with fever, bilateral proptosis, and abdominal distention. He was diagnosed with left suprarenal neuroblastoma (10 × 10 × 8 cm) stage IV with metastasis to skull, skull base and squamous temporal bone plus bilateral femoral, and tibial and bone marrow lesions. Treatment was commenced in the form of a high-risk neuroblastoma chemotherapy protocol in four cycles (cisplatin, etoposide (VP16), adriamycin, cyclophosphamide, ifosfamide, and carboplatin). The patient responded well to treatment, the symptoms resolved, and the tumor reduced in size. He underwent surgical resection of residual tumor a few months later followed by a fifth cycle of chemotherapy and thirteen sessions of localized intensity-modulated radiotherapy (IMRT) radiation protocol to the abdomen and stem cell transplantation was carried out. There was no evidence of residual disease in follow-up CT brain scans in the temporal bone, middle ear, and external auditory canal. Later, at the age of 6 years, he was referred to the ENT clinic with a right ear canal mass associated with recurrent infection and hearing loss. Examination showed a mass originating from the right external auditory meatus and obstructing the ear canal. Computed tomography (CT) of temporal bone with 1 mm slice thickness showed a soft tissue mass in the right external auditory canal with bony remodeling, soft tissue in the mesotympanum, hypotympanum, and middle ear cavity extending into Prussak's space and minimal erosion of the scutum. The ossicles appeared intact. There was also soft tissue seen in the sinus tympani (Figures 1–3).

**Figure 4:**
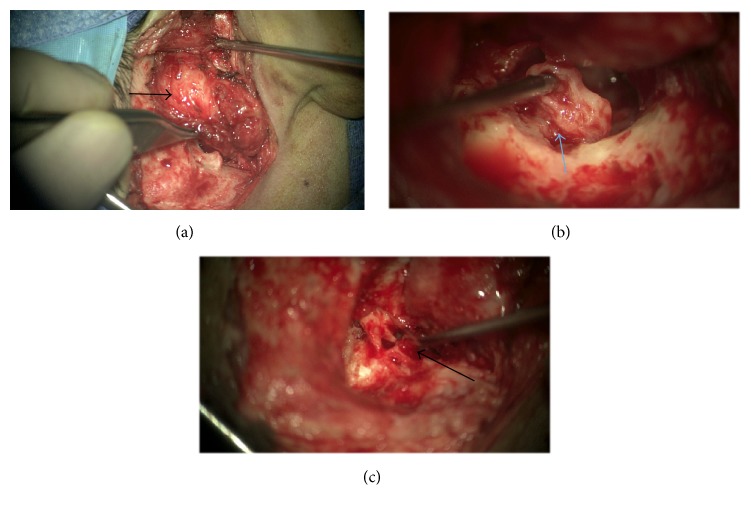
Intraoperative images. (a) Showing lesion filling ear canal and extending out onto the squamous temporal bone (black arrow). (b) Showing disease on the facial nerve (blue arrow). (c) Postremoval of posterior canal wall, showing ossicles and disease on the facial nerve (black arrow).

He underwent right ear examination under general anesthesia with multiple biopsies and partial debulking of the lesion. Histopathology showed ganglioneuroma with no malignancy or neuroblastic elements seen. Based on this histopathology, the patient was then taken to theatre for exploration and excision of the tumor with facial nerve monitoring. The tumor was found to be present above the ear canal and beneath the temporalis muscle extending forward almost to the parotid and onto the zygomatic arch passing down the ear canal to the annulus, causing a degree of bone erosion. The tympanic membrane was normal anteriorly, but posteriorly the drum was displaced laterally by tumor in the middle ear, which extended up into the epitympanum and back into some of the mastoid air cells. There was an extension attached to the vertical portion of the facial nerve as well as the long process of incus and stapes. With the assistance of facial nerve stimulation, the tumor was freed from the facial nerve without damage. The perineural lesion was removed from the facial recess and sinus tympani completely (Figure 4). It was necessary to take down the posterior wall of the ear canal and remove the incus and head of malleus to fully expose the disease. An incus transposition was then performed to optimise hearing and the drum and cavity were grafted with temporalis fascia. One-month follow-up revealed normal facial nerve function with a clean mastoid cavity. The patient will remain on long-term follow-up.

## 3. Final Histopathology

**Figure 5:**
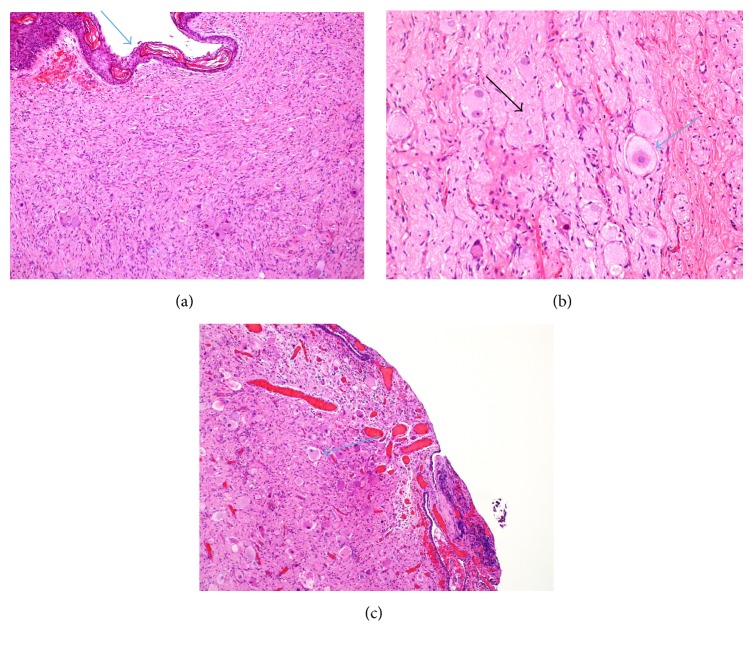
Hematoxylin and eosin stain (H&E stain). (a) Ear canal ganglioneuroma (10x). Showing (arrow) surface squamous epithelium of the external auditory canal and the ganglioneuroma beneath the surface. (b) High power (40x) showing ganglion cells (blue arrow) without rim of satellite cells among Schwannian stroma (black arrow). (c) (10x) Epitympanum showing ganglion cells (arrow).

Ganglioneuromas show bundles of longitudinal and transverse bundles of Schwann cells that irregularly crisscross each other. The ganglion cells are large and dysmorphic, often without satellite cells (maturing rather than mature ganglion cells) and they tend to cluster in some foci more than others. There are no neuroblasts seen in the current specimen, which was submitted entirely (Figure 5).

**Figure 6:**
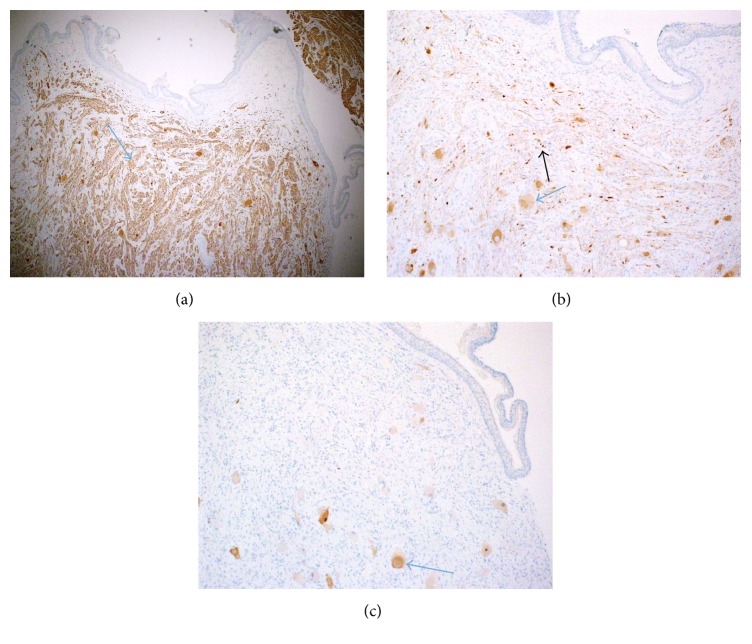
(a) Neurofilaments protein immunostain, showing bundles of axons (arrow). (b) Synaptophysin immunostain, showing cytoplasm of ganglion cells (blue arrow) and also axons (black arrow). (c) Neuronal nuclear immunostain, showing the ganglion cells cytoplasm and nuclei (arrow).

Immunohistochemistry staining used neurofilaments protein immunostain (showing bundles of axons), synaptophysin immunostain (showing cytoplasm of ganglion cells and also axons), and neuronal nuclear immunostain (showing the ganglion cells' cytoplasm and nuclei) (Figure 6). However, since this patient had a remote history of neuroblastoma, this most likely represents an old metastasis with maturation, which is one of the theories about ganglioneuromas representing the maturation of metastatic neuroblastoma.

## 4. Discussion

Ganglioneuroma is a benign, slow-growing tumor originating from the sympathetic chain. The most common sites of presentation are the posterior mediastinum and retroperitoneum. Rarely do they present in the head and neck region [3–7]. There is one case report of ganglioneuroma confined to the external auditory canal with no middle ear involvement in 1975 and another case reporting involvement of the middle ear only [8, 9]. There are multiple studies reporting the same disease in the internal auditory meatus and cervical, parapharyngeal, and retropharyngeal region, presenting with mass effect on the adjacent structures [3–7]. Our patient presented with a mass occluding the external auditory canal causing recurrent infection and hearing loss.

Neuroblastoma, ganglioneuroblastoma, and ganglioneuroma are tumors of varying maturity with malignant or premalignant tendency with immature cells in neuroblastoma and ganglioneuroblastoma and benign histopathology with ganglioneuroma [1, 2]. Our patient had a previous history of neuroblastoma of the abdomen with evident metastasis to squamous part of the temporal bone, although there was no evidence of residual disease in follow-up CT brain scans in the temporal bone, middle ear, and external auditory canal. However, in theory, microscopic cells could mature following chemotherapy and present as ganglioneuroma years later [10].

Preferred imaging in cases of ganglioneuroma is CT scan, to assess the extent of disease, origin, calcification, local extension, and vascular enhancement. CT scan with contrast shows low attenuation and calcification, while T2 MRI scans show heterogeneity with high signal intensity [2, 11].

Clinically significant catecholamine secreting ganglioneuroma is rare [1, 3, 4]. Our patient had no symptoms to indicate a catecholamine secreting tumor and urine levels were not assessed.

Ganglioneuroma is exceedingly rare in the external auditory canal, with only one reported case and no reports of examples involving both the external auditory canal and middle ear [8, 9]. CT scanning in more than one plane is important to detect middle ear involvement. Surgical resection of the tumor is the treatment of choice, while “watchful waiting” is not recommended as there is a reported incidence of malignant transformation [3–7]. There was a significant risk of facial nerve injury in our case, which was minimized with facial nerve monitoring. However, facial nerve scarification would have to be considered in tumors directly invading the nerve.

## Additional Points

*Summary*. (i) Ganglioneuromas are rare benign mature tumors thought to originate from sympathetic ganglions. (ii) Highest incidence of ganglioneuromas occurs in the retroperitoneum, adrenal medulla, and posterior mediastinum. (iii) Ganglioneuroma is a rare disease entity in the ear structures. (iv) Preferred imaging in case of ganglioneuroma is CT. (v) Clinically significant catecholamine secreting ganglioneuroma is rare. (vi) Surgical resection of the tumor is the treatment of choice, while “watchful waiting” is not recommended as there is a reported incidence of malignant transformation. (vii) Facial nerve monitoring intraoperatively is recommended. (viii) Facial nerve scarification should be considered in tumor invading the facial nerve. (ix) In treated cases of neuroblastoma with metastases, ganglioneuroma should be considered in the differential diagnosis of patients presenting with recurrent ear discharge and decreased hearing.
